# Methyl 2-(1a,4a-dimethyl-2,8-dioxo-2,3,4,4a,5,6,7,8-octa­hydro-1a*H*-1-oxacyclo­propa[*d*]naphthalen-7-yl)acrylate

**DOI:** 10.1107/S1600536812000086

**Published:** 2012-01-14

**Authors:** Mohamed Tebbaa, Ahmed Benharref, Jean Claude Daran, Fouad Mellouki, Moha Berraho

**Affiliations:** a Laboratoire de Chimie Biomoleculaire, Substances Naturelles et Réactivité, URAC16, Faculté des Sciences Semlalia, BP 2390 Bd My Abdellah, 40000 Marrakech, Morocco; b Laboratoire de Chimie de Coordination, 205 route de Narbonne, 31077 Toulouse Cedex 04, France

## Abstract

The title compound, C_16_H_20_O_5_, was synthesized from ilicic acid [2-(8-hy­droxy-4a,8-dimethyl­deca­hydro­naphthalen-2-yl)acrylic acid], which was isolated from the chloro­form extract of the aerial part of *Inula viscose* (L) Aiton [or *Dittrichia viscosa­* (L) Greuter]. The molecule is built up from two fused six-membered rings, the epoxidized six-membered ring adopts a half-chair conformation while the other ring displays a perfect chair conformation. The crystal structure features C—H⋯O hydrogen bonds.

## Related literature

For medicinal background to *Inula Viscosa­* (L) Aiton [or *Dittrichia Viscosa­* (L) Greuter], see: Shtacher & Kasshman (1970 ▶); Chiappini *et al.* (1982 ▶); Azoulay *et al.* (1986 ▶); Bohlman *et al.* (1977 ▶); Ceccherelli *et al.* (1988 ▶); Geissman & Toribio (1967 ▶) For the synthesis, see: Barrero *et al.* (2009 ▶); Tebbaa *et al.* (2011 ▶). For conformational analysis, see: Cremer & Pople (1975 ▶).
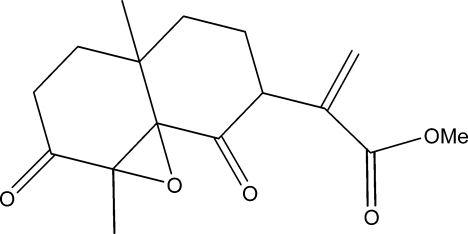


## Experimental

### 

#### Crystal data

C_16_H_20_O_5_
*M* *_r_* = 292.32
Orthorhombic, 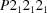

*a* = 8.8626 (3) Å
*b* = 9.4552 (3) Å
*c* = 17.4080 (5) Å
*V* = 1458.75 (8) Å^3^
*Z* = 4
Mo *K*α radiation
μ = 0.10 mm^−1^
*T* = 180 K
0.45 × 0.33 × 0.12 mm

#### Data collection

Oxford Diffraction Xcalibur Sapphire1 long nozzle diffractometer
Absorption correction: multi-scan (*CrysAlis PRO*; Oxford Diffraction, 2010 ▶) *T*_min_ = 0.650, *T*_max_ = 1.000
33985 measured reflections
1716 independent reflections
1638 reflections with *I* > 2σ(*I*)
*R*_int_ = 0.040

#### Refinement

*R*[*F* ^2^ > 2σ(*F* ^2^)] = 0.028
*wR*(*F* ^2^) = 0.075
*S* = 1.06
1716 reflections
193 parameters
H-atom parameters constrained
Δρ_max_ = 0.20 e Å^−3^
Δρ_min_ = −0.15 e Å^−3^

### 

Data collection: *CrysAlis PRO* (Oxford Diffraction, 2010 ▶); cell refinement: *CrysAlis PRO*; data reduction: *CrysAlis PRO*; program(s) used to solve structure: *SHELXS97* (Sheldrick, 2008 ▶); program(s) used to refine structure: *SHELXL97* (Sheldrick, 2008 ▶); molecular graphics: *ORTEP-3 for Windows* (Farrugia, 1997 ▶) and *PLATON* (Spek, 2009 ▶); software used to prepare material for publication: *WinGX* (Farrugia, 1999 ▶).

## Supplementary Material

Crystal structure: contains datablock(s) I, global. DOI: 10.1107/S1600536812000086/bt5772sup1.cif

Structure factors: contains datablock(s) I. DOI: 10.1107/S1600536812000086/bt5772Isup2.hkl

Supplementary material file. DOI: 10.1107/S1600536812000086/bt5772Isup3.cml

Additional supplementary materials:  crystallographic information; 3D view; checkCIF report

## Figures and Tables

**Table 1:** Hydrogen-bond geometry (Å, °)

*D*—H⋯*A*	*D*—H	H⋯*A*	*D*⋯*A*	*D*—H⋯*A*
C3—H3*A*⋯O5^i^	0.97	2.57	3.492 (2)	158
C5—H5*A*⋯O4^ii^	0.97	2.50	3.337 (2)	145
C7—H7⋯O3^ii^	0.98	2.54	3.3321 (19)	138
C7—H7⋯O4^ii^	0.98	2.54	3.3877 (19)	145

Symmetry codes: (i) 
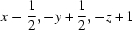
; (ii) 
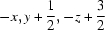
.

**Fig. 1.**
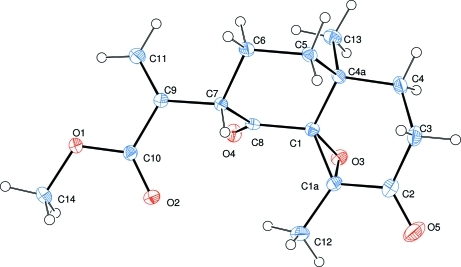
: Molecular structure of the title compound with the atom-labelling scheme. Displacement ellipsoids are drawn at the 30% probability level. H atoms are represented as small spheres of arbitrary radii.

**Fig. 2.**
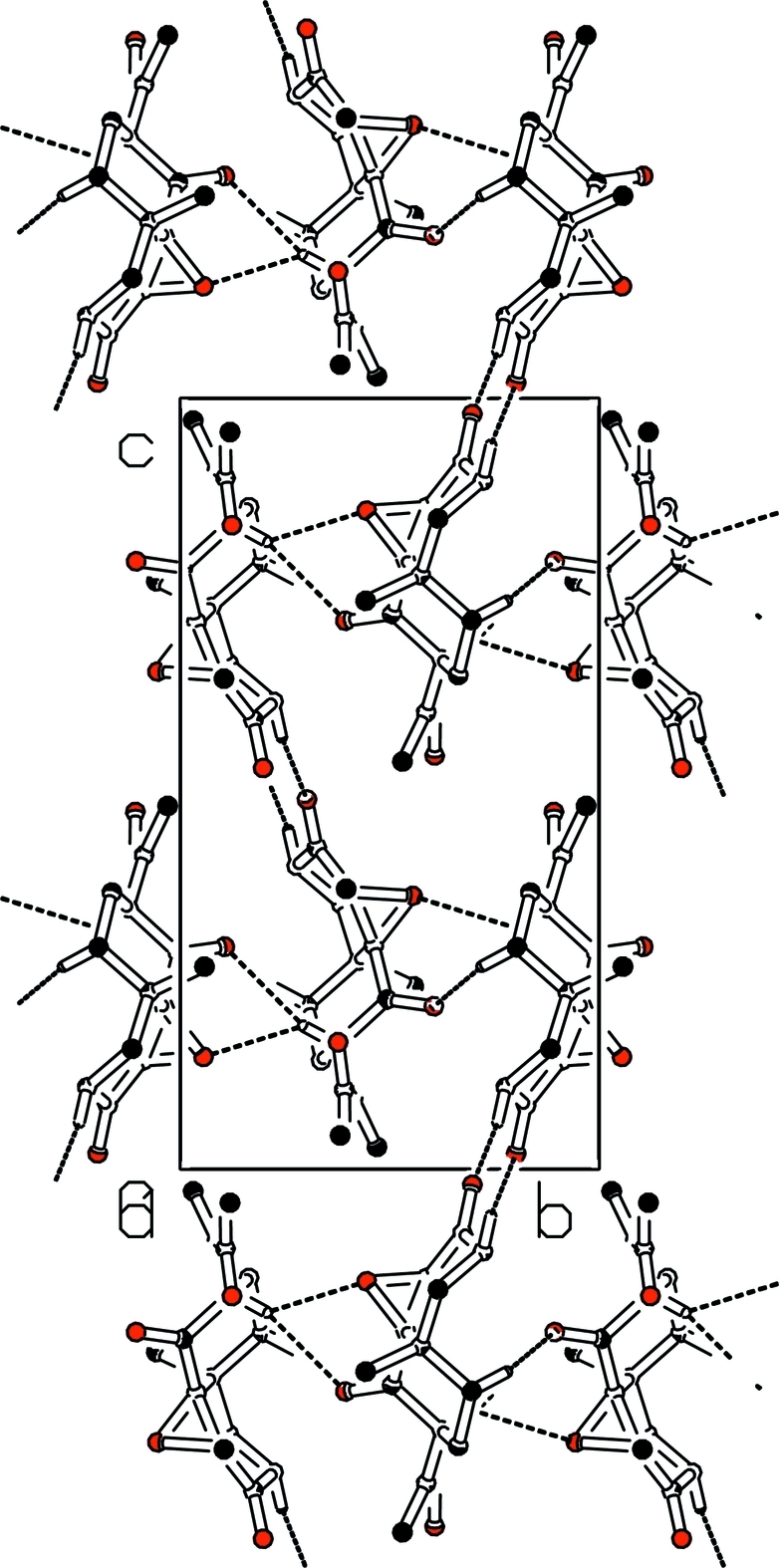
: packing view showing the C–H···O hydrogen bonds as dashed lines. H atoms not involved in hydrogen bonding have been omitted for clarity.

**Fig. 3.**
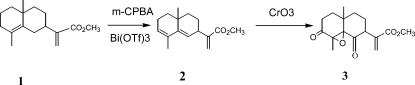
: Synthesis of the title compound.

## supplementary crystallographic information

### Comment

Our work lies within the framework of the evaluation of medicinals plants and in particular, *Inula Viscosa (L) Aiton or Dittrichia Viscosa (L) Greuter]*. This plant is widespread in Mediterranean area and extends to the Atlantic cost of Morocco. It is a well known medicinal plant (Shtacher & Kasshman, 1970; Chiappini *et al.*, 1982) and has some pharmacological activities (Azoulay *et al.*, 1986). This plant has been the subject of chemical investigation in terms of isolating sesquiterpene lactones (Bohlman *et al.*, 1977), sesquiterpene acids (Ceccherelli *et al.*, 1988; Geissman & Toribio, 1967). The ilicic acid is one of the main components of the dichloromethane extract of the Inula Viscosa (*L*) Aiton or Dittrichia Viscosa (*L*) Greuter]. The literature reports one article on the transformation of the ilicic acid (Barrero *et al.*, 2009). In order to prepare products with high added value, that can be used in the pharmacologycal industry, we have studied the reactivity of this sesquiterpene acid. Thus, from this acid, we have prepared by the method of Barrero *et al.* (2009), 2-(4a,8-Dimethyl-1, 2,3,4,4 a,5,6,7-octahydro naphthalen-2-yl)-acrylic acid methyl ester(1) (Figure 3). The epoxidation of this sesquiterpene by metachloroperbenzoic acid (mCPBA), followed by the opening of the epoxide, obtained by Bi(OTf)_3_ (Tebbaa *et al.*, 2011), leads to the compound (2) with a yield of 40%. The oxidation of the latter with chromic anhydride (CrO_3_) leads to the title compound with a yield of 60%. The crystal structure of the title compound is escribed herein. The molecule is built up from two fused six-membered rings. The molecular structure (Fig. 1), shows that the two rings adopt different conformations. A perfect chair conformation for the first ring (C1, C4a···C8) as indicated by Cremer & Pople (1975) puckering parameters Q(T)= 0.5561 (19)Å and spherical polar angle θ = 178.35 (18)° with φ = 245 (6)°. While the second ring (C1, C4a···C1a) displays a half chair conformation with Q(T) = 0.4303 (19), θ = 47,5(2)° and φ = 225,8(3). The crystal structure is stabilized by intermolecular C—H···O hydrogen bonds. (Table 1, Figure 2).

### Experimental

To a solution of 1 g (4 mmol) of 2-(4a,8-Dimethyl-2, 3,4,4a,5,6- hexahydro-naphthalene-2-yl)-acrylic acid methyl ester (2) dissolved in 20 ml acetone is added in small portions three equivalents of chromic anhydride (CrO_3_) at 0° C. The reaction mixture is left stirring for 1 h, then treated with 20 ml of cold water and extracted three times with 30 ml of ethyl acetate. The organic phases are combined, dried over sodium sulfate and concentrated under reduced pressure. The residue obtained is chromatographed on silica gel eluting with hexane-ethyl acetate (98–2) allowed the isolation in pure the 2 - (1a, 4a-dimethyl-2, 8 -dioxo- octahydro-1-oxa-cycloprop [*d*] naphthalene-7-yl)-acrylic acid methyl, with a yield of 60% (70 mg, 2.4 mmol). The title compound was recrystallized from its dichloromethane solution at room temperature.

### Refinement

All H atoms attached to C atoms were fixed geometrically and treated as riding with C—H = 0.96 Å (methyl), 0.97 Å (methylene), 0.98Å (methine) and 0.93 Å (C=CH_2_) with *U*_iso_(H) = 1.2*U*_eq_(C) or *U*_iso_(H) = 1.5*U*_eq_(C_methyl_). In the absence of significant anomalous scatterers, the absolute configuration could not be reliably determined and Friedel pairs were merged and any references to the Flack parameter were removed.

### Crystal data

**Table:**

C_16_H_20_O_5_	*F*(000) = 624
*M**_r_* = 292.32	*D*_x_ = 1.331 Mg m^−^^3^
Orthorhombic, *P*2_1_2_1_2_1_	Mo *K*α radiation, λ = 0.71073 Å
Hall symbol: P 2ac 2ab	Cell parameters from 33985 reflections
*a* = 8.8626 (3) Å	θ = 3.2–26.4°
*b* = 9.4552 (3) Å	µ = 0.10 mm^−^^1^
*c* = 17.4080 (5) Å	*T* = 180 K
*V* = 1458.75 (8) Å^3^	Prism, colourless
*Z* = 4	0.45 × 0.33 × 0.12 mm

### Data collection

**Table:**

Oxford Diffraction Xcalibur Sapphire1 long nozzle diffractometer	1716 independent reflections
Radiation source: fine-focus sealed tube	1638 reflections with *I* > 2σ(*I*)
graphite	*R*_int_ = 0.040
Detector resolution: 8.2632 pixels mm^-1^	θ_max_ = 26.4°, θ_min_ = 3.2°
ω scans	*h* = −11→11
Absorption correction: multi-scan (CrysAlis PRO; Oxford Diffraction, 2010)	*k* = −11→11
*T*_min_ = 0.650, *T*_max_ = 1.000	*l* = −21→21
33985 measured reflections	

### Refinement

**Table:**

Refinement on *F*^2^	Primary atom site location: structure-invariant direct methods
Least-squares matrix: full	Secondary atom site location: difference Fourier map
*R*[*F*^2^ > 2σ(*F*^2^)] = 0.028	Hydrogen site location: inferred from neighbouring sites
*wR*(*F*^2^) = 0.075	H-atom parameters constrained
*S* = 1.06	*w* = 1/[σ^2^(*F*_o_^2^) + (0.0462*P*)^2^ + 0.2444*P*] where *P* = (*F*_o_^2^ + 2*F*_c_^2^)/3
1716 reflections	(Δ/σ)_max_ < 0.001
193 parameters	Δρ_max_ = 0.20 e Å^−^^3^
0 restraints	Δρ_min_ = −0.15 e Å^−^^3^

### Special details

**Table:**

Experimental. Empirical absorption correction using spherical harmonics, implemented in SCALE3 ABSPACK scaling algorithm. CrysAlisPro (Oxford Diffraction, 2010)
Geometry. All s.u.'s (except the s.u. in the dihedral angle between two l.s. planes) are estimated using the full covariance matrix. The cell s.u.'s are taken into account individually in the estimation of s.u.'s in distances, angles and torsion angles; correlations between s.u.'s in cell parameters are only used when they are defined by crystal symmetry. An approximate (isotropic) treatment of cell s.u.'s is used for estimating s.u.'s involving l.s. planes.
Refinement. Refinement of *F*^2^ against ALL reflections. The weighted *R*-factor *wR* and goodness of fit *S* are based on *F*^2^, conventional *R*-factors *R* are based on *F*, with *F* set to zero for negative *F*^2^. The threshold expression of *F*^2^ > 2σ(*F*^2^) is used only for calculating *R*-factors(gt) *etc*. and is not relevant to the choice of reflections for refinement. *R*-factors based on *F*^2^ are statistically about twice as large as those based on *F*, and *R*- factors based on ALL data will be even larger.

### Fractional atomic coordinates and isotropic or equivalent isotropic displacement parameters (Å^2^)

**Table:**

	*x*	*y*	*z*	*U*_iso_*/*U*_eq_	
C1	−0.13339 (18)	0.03809 (16)	0.70987 (9)	0.0207 (3)	
C1A	−0.05450 (19)	0.08964 (18)	0.64052 (9)	0.0239 (3)	
C2	−0.1449 (2)	0.1740 (2)	0.58274 (10)	0.0299 (4)	
C3	−0.2984 (2)	0.2227 (2)	0.60642 (10)	0.0330 (4)	
H3A	−0.3578	0.2417	0.5607	0.040*	
H3B	−0.2889	0.3106	0.6347	0.040*	
C4	−0.3818 (2)	0.1157 (2)	0.65598 (10)	0.0304 (4)	
H4A	−0.4002	0.0313	0.6257	0.036*	
H4B	−0.4790	0.1549	0.6702	0.036*	
C4A	−0.29707 (17)	0.07384 (17)	0.72924 (9)	0.0220 (3)	
C5	−0.29623 (18)	0.19549 (17)	0.78766 (9)	0.0233 (3)	
H5A	−0.2565	0.2797	0.7631	0.028*	
H5B	−0.3992	0.2154	0.8031	0.028*	
C6	−0.20299 (18)	0.16391 (19)	0.85886 (9)	0.0244 (4)	
H6A	−0.2494	0.0868	0.8870	0.029*	
H6B	−0.2025	0.2465	0.8919	0.029*	
C7	−0.03943 (17)	0.12379 (16)	0.83876 (8)	0.0194 (3)	
H7	0.0067	0.2064	0.8142	0.023*	
C8	−0.04044 (17)	0.00594 (16)	0.78003 (9)	0.0204 (3)	
C9	0.05601 (18)	0.08709 (18)	0.90730 (9)	0.0224 (3)	
C10	0.22089 (18)	0.10826 (18)	0.89680 (9)	0.0221 (3)	
C11	0.0043 (2)	0.0305 (3)	0.97121 (11)	0.0409 (5)	
H11A	0.0709	0.0037	1.0098	0.049*	
H11B	−0.0989	0.0173	0.9777	0.049*	
C12	0.1137 (2)	0.1040 (2)	0.63642 (10)	0.0322 (4)	
H12A	0.1479	0.0766	0.5863	0.048*	
H12B	0.1594	0.0442	0.6744	0.048*	
H12C	0.1415	0.2006	0.6460	0.048*	
C13	−0.3722 (2)	−0.05753 (19)	0.76273 (11)	0.0314 (4)	
H13A	−0.3246	−0.0817	0.8105	0.047*	
H13B	−0.3620	−0.1348	0.7273	0.047*	
H13C	−0.4773	−0.0388	0.7715	0.047*	
C14	0.4577 (2)	0.1183 (3)	0.95628 (11)	0.0376 (5)	
H14A	0.4832	0.2048	0.9305	0.056*	
H14B	0.4952	0.0395	0.9272	0.056*	
H14C	0.5025	0.1178	1.0065	0.056*	
O1	0.29605 (13)	0.10753 (16)	0.96328 (7)	0.0318 (3)	
O2	0.28032 (14)	0.12332 (15)	0.83537 (7)	0.0307 (3)	
O3	−0.11162 (14)	−0.05477 (12)	0.64566 (6)	0.0263 (3)	
O4	0.02356 (14)	−0.10571 (13)	0.78860 (7)	0.0298 (3)	
O5	−0.09164 (19)	0.19766 (18)	0.52039 (8)	0.0492 (4)	

### Atomic displacement parameters (Å^2^)

**Table:**

	*U*^11^	*U*^22^	*U*^33^	*U*^12^	*U*^13^	*U*^23^
C1	0.0221 (8)	0.0195 (7)	0.0205 (7)	0.0005 (6)	0.0014 (6)	−0.0044 (6)
C1A	0.0274 (8)	0.0231 (8)	0.0211 (7)	0.0002 (7)	0.0024 (6)	−0.0017 (6)
C2	0.0388 (10)	0.0275 (8)	0.0235 (8)	0.0001 (8)	−0.0015 (7)	0.0006 (7)
C3	0.0358 (10)	0.0372 (10)	0.0261 (8)	0.0086 (9)	−0.0073 (8)	0.0024 (8)
C4	0.0239 (8)	0.0380 (10)	0.0293 (8)	0.0020 (8)	−0.0073 (7)	−0.0046 (8)
C4A	0.0181 (7)	0.0245 (8)	0.0234 (7)	0.0004 (6)	−0.0007 (6)	−0.0025 (7)
C5	0.0192 (7)	0.0251 (8)	0.0255 (7)	0.0042 (6)	0.0000 (6)	−0.0029 (7)
C6	0.0205 (8)	0.0291 (9)	0.0235 (8)	0.0042 (7)	0.0015 (7)	−0.0052 (7)
C7	0.0184 (7)	0.0201 (7)	0.0198 (7)	0.0001 (6)	0.0003 (6)	0.0001 (6)
C8	0.0161 (7)	0.0225 (7)	0.0226 (7)	−0.0004 (6)	0.0049 (6)	0.0004 (6)
C9	0.0212 (8)	0.0242 (8)	0.0218 (7)	0.0006 (7)	0.0005 (6)	−0.0003 (6)
C10	0.0215 (8)	0.0232 (8)	0.0215 (7)	0.0022 (7)	−0.0012 (6)	0.0009 (7)
C11	0.0256 (9)	0.0678 (14)	0.0294 (9)	−0.0027 (9)	−0.0007 (8)	0.0167 (10)
C12	0.0284 (9)	0.0370 (10)	0.0314 (8)	−0.0018 (9)	0.0083 (7)	−0.0012 (8)
C13	0.0262 (9)	0.0300 (9)	0.0379 (9)	−0.0058 (8)	0.0026 (8)	−0.0030 (8)
C14	0.0194 (8)	0.0599 (12)	0.0336 (9)	−0.0015 (9)	−0.0043 (7)	0.0025 (10)
O1	0.0201 (6)	0.0533 (8)	0.0221 (6)	−0.0018 (6)	−0.0024 (5)	0.0008 (6)
O2	0.0256 (6)	0.0437 (8)	0.0226 (6)	0.0003 (6)	0.0034 (5)	0.0049 (6)
O3	0.0327 (6)	0.0227 (6)	0.0235 (6)	0.0004 (5)	0.0021 (5)	−0.0059 (5)
O4	0.0329 (6)	0.0234 (6)	0.0331 (6)	0.0086 (5)	−0.0033 (5)	−0.0013 (5)
O5	0.0603 (9)	0.0587 (10)	0.0286 (7)	0.0110 (8)	0.0104 (7)	0.0139 (7)

### Geometric parameters (Å, °)

**Table:**

C1—O3	1.4344 (19)	C6—H6B	0.9700
C1—C1A	1.478 (2)	C7—C9	1.503 (2)
C1—C8	1.504 (2)	C7—C8	1.512 (2)
C1—C4A	1.527 (2)	C7—H7	0.9800
C1A—O3	1.459 (2)	C8—O4	1.208 (2)
C1A—C12	1.499 (2)	C9—C11	1.317 (2)
C1A—C2	1.513 (2)	C9—C10	1.486 (2)
C2—O5	1.204 (2)	C10—O2	1.2005 (19)
C2—C3	1.495 (3)	C10—O1	1.3354 (19)
C3—C4	1.521 (3)	C11—H11A	0.9300
C3—H3A	0.9700	C11—H11B	0.9300
C3—H3B	0.9700	C12—H12A	0.9600
C4—C4A	1.532 (2)	C12—H12B	0.9600
C4—H4A	0.9700	C12—H12C	0.9600
C4—H4B	0.9700	C13—H13A	0.9600
C4A—C13	1.525 (2)	C13—H13B	0.9600
C4A—C5	1.535 (2)	C13—H13C	0.9600
C5—C6	1.519 (2)	C14—O1	1.442 (2)
C5—H5A	0.9700	C14—H14A	0.9600
C5—H5B	0.9700	C14—H14B	0.9600
C6—C7	1.539 (2)	C14—H14C	0.9600
C6—H6A	0.9700		
			
O3—C1—C1A	60.11 (10)	C5—C6—H6B	109.2
O3—C1—C8	115.80 (13)	C7—C6—H6B	109.2
C1A—C1—C8	118.09 (13)	H6A—C6—H6B	107.9
O3—C1—C4A	115.81 (12)	C9—C7—C8	111.71 (13)
C1A—C1—C4A	123.84 (14)	C9—C7—C6	113.99 (13)
C8—C1—C4A	112.69 (13)	C8—C7—C6	109.26 (12)
O3—C1A—C1	58.47 (10)	C9—C7—H7	107.2
O3—C1A—C12	115.66 (14)	C8—C7—H7	107.2
C1—C1A—C12	122.65 (15)	C6—C7—H7	107.2
O3—C1A—C2	110.53 (14)	O4—C8—C1	122.29 (15)
C1—C1A—C2	117.81 (15)	O4—C8—C7	123.90 (15)
C12—C1A—C2	116.54 (16)	C1—C8—C7	113.78 (13)
O5—C2—C3	123.23 (18)	C11—C9—C10	120.07 (15)
O5—C2—C1A	119.32 (18)	C11—C9—C7	124.64 (15)
C3—C2—C1A	117.45 (15)	C10—C9—C7	115.12 (14)
C2—C3—C4	113.19 (16)	O2—C10—O1	123.63 (15)
C2—C3—H3A	108.9	O2—C10—C9	123.85 (15)
C4—C3—H3A	108.9	O1—C10—C9	112.52 (14)
C2—C3—H3B	108.9	C9—C11—H11A	120.0
C4—C3—H3B	108.9	C9—C11—H11B	120.0
H3A—C3—H3B	107.8	H11A—C11—H11B	120.0
C3—C4—C4A	113.97 (14)	C1A—C12—H12A	109.5
C3—C4—H4A	108.8	C1A—C12—H12B	109.5
C4A—C4—H4A	108.8	H12A—C12—H12B	109.5
C3—C4—H4B	108.8	C1A—C12—H12C	109.5
C4A—C4—H4B	108.8	H12A—C12—H12C	109.5
H4A—C4—H4B	107.7	H12B—C12—H12C	109.5
C13—C4A—C1	108.59 (14)	C4A—C13—H13A	109.5
C13—C4A—C4	108.35 (14)	C4A—C13—H13B	109.5
C1—C4A—C4	109.80 (13)	H13A—C13—H13B	109.5
C13—C4A—C5	111.05 (13)	C4A—C13—H13C	109.5
C1—C4A—C5	107.90 (12)	H13A—C13—H13C	109.5
C4—C4A—C5	111.11 (13)	H13B—C13—H13C	109.5
C6—C5—C4A	113.32 (13)	O1—C14—H14A	109.5
C6—C5—H5A	108.9	O1—C14—H14B	109.5
C4A—C5—H5A	108.9	H14A—C14—H14B	109.5
C6—C5—H5B	108.9	O1—C14—H14C	109.5
C4A—C5—H5B	108.9	H14A—C14—H14C	109.5
H5A—C5—H5B	107.7	H14B—C14—H14C	109.5
C5—C6—C7	112.04 (13)	C10—O1—C14	114.97 (13)
C5—C6—H6A	109.2	C1—O3—C1A	61.42 (10)
C7—C6—H6A	109.2		

### Hydrogen-bond geometry (Å, °)

**Table:**

*D*—H···*A*	*D*—H	H···*A*	*D*···*A*	*D*—H···*A*
C3—H3A···O5^i^	0.97	2.57	3.492 (2)	158
C5—H5A···O4^ii^	0.97	2.50	3.337 (2)	145
C7—H7···O3^ii^	0.98	2.54	3.3321 (19)	138
C7—H7···O4^ii^	0.98	2.54	3.3877 (19)	145

Symmetry codes: (i) *x*−1/2, −*y*+1/2, −*z*+1; (ii) −*x*, *y*+1/2, −*z*+3/2.
